# Migrant workers’ occupation and healthcare-seeking preferences for TB-suspicious symptoms and other health problems: a survey among immigrant workers in Songkhla province, southern Thailand

**DOI:** 10.1186/1472-698X-12-22

**Published:** 2012-10-02

**Authors:** Tinzar Naing, Alan Geater, Petchawan Pungrassami

**Affiliations:** 1 National Tuberculosis Programme, Department of Health, Nay Pyi Taw, Myanmar; 2 Epidemiology Unit, Faculty of Medicine, Prince of Songkla University, Hatyai, Songkhla, Thailand; 3 TB Centre 12, Amphoe Muang Yala, Ministry of Public Health, , Thailand

## Abstract

**Background:**

Much of the unskilled and semi-skilled workforce in Thailand comprises migrant workers from neighbouring countries. While, in principle, healthcare facilities in the host country are open to those migrants registered with the Ministry of Labour, their actual healthcare-seeking preferences and practices, as well as those of unregistered migrants, are not well documented. This study aimed to describe the patterns of healthcare-seeking behaviours of immigrant workers in Thailand, emphasizing healthcare practices for TB-suspicious symptoms, and to identify the role of occupation and other factors influencing these behaviours.

**Methods:**

A survey was conducted among 614 immigrant factory workers (FW), rubber tappers (RT) and construction workers (CW), in which information was sought on socio-demography, history of illness and related healthcare-seeking behaviour. Mixed effects logistic regression modeling was employed in data analysis.

**Results:**

Among all three occupations, self-medication was the most common way of dealing with illnesses, including the development of TB-suspicious symptoms, for which inappropriate drugs were used. Only for GI symptoms and obstetric problems did migrant workers commonly seek healthcare at modern healthcare facilities. For GI illness, FW preferred to attend the in-factory clinic and RT a private facility over government facilities owing to the quicker service and greater convenience. For RT, who were generally wealthier, the higher cost of private treatment was not a deterrent. CW preferentially chose a government healthcare facility for their GI problems. For obstetric problems, including delivery, government facilities were utilized by RT and CW, but most FW returned to their home country. After adjusting for confounding, having legal status in the country was associated with overall greater use of government facilities and being female and being married with use of both types of modern healthcare facility. One-year estimated period prevalence of TB-suspicious symptoms was around 6% among FW but around 27% and 30% in RT and CW respectively. However, CW were the least likely to visit a modern healthcare facility for these symptoms.

**Conclusions:**

Self medication is the predominant mode of healthcare seeking among these migrant workers. When accessing a modern healthcare facility the choice is influenced by occupation and its attendant lifestyle and socioeconomic conditions. Utilization of modern facilities could be improved by reducing the current barriers by more complete registration coverage and better provision of healthcare information, in which local vendors of the same ethnicity could play a useful role. Active surveillance for TB among migrant workers, especially CW, may lead to better TB control.

## Background

We live in a highly mobile world. It is an unavoidable consequence of unequal wealth among the world’s nations that migrant workers cross the international borders to gain better economic outcome. The most socially significant migrations can be found within or between developing countries in Asia and Africa [1].

Trans-border migration is a well-known phenomenon in Thailand. Over the past 30 years, Thailand has gradually transitioned from a labour-exporting to a labour-importing country, and hosted hundreds of thousands of nationals from neighbouring countries, resulting in a large influx of low-skilled migrant workers [1]. It is estimated that there are more than 2 million migrant workers from the Greater Mekong Sub-region (GMS) countries in Thailand [2]. Regionalisation is also the other main reason causing migratory consequences for Thailand and its neighbouring countries. Border area travel into Thailand has also risen with the creation of border passes.

Transnational migrant workers are commonly surrounded by difficult and exploitative circumstances, which may be a result of their terms of employment and often precarious legal status [3]. Due to the nature of being migrants, they are likely to experience specific challenges in relation to health. Migration itself also has a major impact on access to and utilization of health services by migrant and host population [4]. There are many barriers to access health services for migrants, such as the fact that migrants need documents to be able to get healthcare services without fear [5].

There are also numerous job opportunities for migrants in Thailand, primarily in sectors that local people have abandoned, such as fishing, construction, factories, domestic works, agriculture (mostly old rubber plantations), and entertainment (principally sex-service) [1,3]. According to their jobs, migrant workers have different life styles, and in response to these, there may be different healthcare-seeking behaviours mediated by a variety of other factors, including convenience, cultural familiarity, consideration of language and legal status [3]. Even though registered migrants are supposedly entitled to the same health services as the local people, they may also have hidden problems restricting their access to an adequate level of healthcare.

Poor socio-economic status of migrant workers in Thailand creates a situation where the health risks may be increased. The diseases they suffer from are more life-threatening than those suffered by the Thai population [1]. Many migrants are likely to stay in the environments characterized by overcrowded, substandard housing, poor sanitation and lack of access to medical services. Such conditions may not only restrict their access to healthcare services but also favour the spread of communicable diseases within their communities. Indeed, tuberculosis was identified in the nation-wide health examination of migrants, conducted in 2004 by the Thai Ministry of Public Health, as the most common disease of concern among migrants [3].

Increased probability of becoming infected with TB and developing active TB are both associated with malnutrition, crowding, poor air circulation and poor sanitation [6]. Over-crowded, insalubrious living conditions of migrants may amplify the ease of spread of infectious diseases among their population [4]. Growing international migration is also thought to be the greatest factor contributing to the rise in cases and changes observed in the epidemiology of TB in the developed world [6].

However, despite the worrisome situation and the wide recognition of TB as a major health issue among migrants, there is still little knowledge on how they seek healthcare when they suffer from TB-suspicious symptoms and other ailments during their stay in Thailand. Migrant workers rarely complain due to the fear of dismissal, arrest, deportation and unfamiliarity with the Thai language and legal system [1] Previous research conducted in Thailand revealed that migrants from neighbouring countries were facing a number of barriers to accessing health services even though their cultural background was similar to that of the host country [7].

In this study, the impact of occupation on the healthcare-seeking behaviour of migrant manual workers in Thailand was assessed. Particular emphasis was placed on the healthcare-seeking behaviours in response to the occurrence of TB-suspicious symptoms.

## Methods

### Study setting and design

The study was set in Songkhla province, Southern Thailand. Songkhla province, situated on the east coast of peninsular Thailand, is the major commercial centre of the southern region with various kinds of factory (rubber processing, wood processing, seafood processing) and many construction sites, and is surrounded by extensive areas of rubber plantation. The area has many migrant workers with diverse cultural systems, beliefs, and healthcare-seeking practices.

Initially, major sites of residence of migrants in the study area were identified with the help of key informants from among the foreign migrant population who were well acquainted with the migrant population within Songkhla province and could provide information on occupations in which migrants were working, places of work and residence, approximate population sizes, and their lifestyle. Several migrant workers from each occupation were informally interviewed in depth to obtain as much information as possible, recorded in field notes, to aid in constructing the questionnaire to be subsequently used to obtain data on healthcare-seeking behaviour, legal status, occupation and other sociodemographic characteristics. The qualitative information collected in this way was also used as an aid to interpreting the quantitative data.

A cross-sectional survey was conducted on non-contrived samples from each of three occupational migrant populations, factory workers, rubber tappers and construction workers, during the period June-November, 2010. Only workers aged 15 to 60 years were recruited.

### Sample size

Sample size was calculated to provide a precision (95% confidence interval) of estimated prevalences within each occupation of between ±5% and ±9% for prevalences between 10% and 40% respectively. Incorporating an anticipated design effect of 1.5, at least two hundred workers per occupation were required.

To identify factors independently associated with various healthcare-seeking behaviours, the sample was considered *in toto*, ie. a minimum of 600 workers. This sample size was sufficient to detect relative differences in behaviour prevalences between occupational groups of between 0.5 and 0.3 times, given that the higher prevalence lay respectively between 50% and 20%, as statistically significant (p < 0.5) with a power of 80%.

### Sampling process

The sampling process comprised two stages. Snowball sampling technique was adopted as the first step to locate workers’ residence camps. This method was adopted because the migrant workers constitute a hard-to-reach population, especially as a large proportion of this population was known to be un-registered, and therefore illegal. Such workers are likely to be socially invisible and wary of outsiders. It has been acknowledged that to attempt a study of hidden populations for whom adequate lists and consequently sampling frames are not readily available, snowball sampling methodology may be the only feasible method available [8].

All sampling was done in the workers’ residential camps and not in the workplaces, to avoid interference and/or restrictions on participation that might be imposed by employers and managers. Sampling strategy differed slightly between rubber tappers and construction workers on the one hand and factory workers on the other.

For rubber tappers and construction workers, the first camp to be included was the one where two key informants from each worker category were working. These two had been working in the study area for more than 10 years. They chose the second camp again, then the workers of the second camp gave more information to key informants to select the third one and in this way, successive camps were selected. To collect data among factory workers, factories were selected from among those that the key informants could contact and that would capture the diversity of product manufactured and workforce size.

Within each camp, respondents were selected randomly (based on room) and one worker from each room (if more than one) randomly chosen for interview. A constant sampling fraction of 30% for rubber tapers and 25% for construction workers was employed in each camp. For factory workers, the required number of workers (200) was divided by the estimated total number of migrant workers at all available factories to give the required sampling fraction.

Finally, migrant workers were interviewed in 14 factory camps, 15 construction camps and 49 rubber plantation camps. The number of rubber plantations camps visited was greater than that of factory and construction camps because only a few workers were resident at each rubber plantation.

### Data collection

Data collection was undertaken between June and November, 2010. Face-to-face interviews following a structured questionnaire were conducted in the migrants’ own language by two interviewers. However, among rubber tappers, some respondents did not well understand the questions and, in these cases, two volunteers of the same ethnicity helped to explain the questions.

Information was sought on socio-demography, legal status, history of illness while in Thailand, healthcare-seeking behaviours for their different kinds of illness in general (namely common cold, aches and pains, GI problems, respiratory problems, urinary problems, obstetric problems, gynaecologic problems and “other health problems”) and for TB-suspicious symptoms in particular. Healthcare-seeking behaviours covered the type of facility or healthcare provider accessed for each type of illness, including government hospital, government health centre, factory clinic, non-governmental organization clinic, private clinic, traditional healer, self medication and “others” – to be specified. Their choices did not need to be mutually exclusive, even within type of illness. Their reasons for attending or not attending a government or a private healthcare facility were also investigated to understand their perception of the difficulties in seeking healthcare.

Only three hours per day were available to collect data among the rubber tappers since they worked at night starting from 11 pm till 10-11 am in the morning, and took rest and slept in the daytime. They were available to be interviewed only in the late afternoon from 4 pm to 7 pm. In the case that the randomly chosen rubber tapper was inebriated, the interview needed to be prolonged or postponed in order to obtain reliable information.

For factory workers and construction workers, an appointment was made 2–3 days prior to the interview. Some factory workers worked two shifts contiguously, and in some cases were not free for a whole week. If such a worker was selected but not available at the appointed time for interview, up to 3 repeat visits were made in order to maximize the response rate.

Construction workers were visited late in the evening from about 6 pm. Most workers stopped work around 5 pm. Those who worked two shifts finished at about 9 pm. If such a worker was selected for interview, the interviewer waited at that camp until the worker returned.

During questioning about TB-suspicious symptoms, those workers who reported using self-medication for such symptoms were shown two kinds of pre-packaged western medicines packets which were readily available at stalls run by vendors of the same nationality as the migrants. They were asked whether they had taken these drugs or not. One combination contained antitussive drug, analgesic agent and dexamethazone. Another combination was made up of acetaminophen, other analgesic agent and sulphadoxine pyrimethamine.

### Data management and statistical analysis

Questionnaires were checked, coded, rechecked and double-entered into a computer using Epidata version 3.1 and transferred into Stata format for analysis using Stata version 7.0 (StataCorp, College Station, Texas) and R version 2.12.1 (R Foundation for Statistical Computing, 2010).

Data were cleaned, recoded as necessary and summarized using tabulation. Sociodemographic characteristics, illness experience and healthcare seeking behaviours were compared across the three occupational groups.

Variables potentially related to the use of a governmental and of a private healthcare facility for illnesses in general were explored in a series of univariate analyses and significance levels examined using chi-square test, Fisher’s exact test, Mann–Whitney test or Kruskal-Wallis test, as appropriate to the type of variable. Variables having a p-value of <0.2 were selected for further inclusion in multivariate mixed effects logistic regression models.

A similar procedure was followed to identify potential predictors of using any healthcare facility for TB-suspicious symptoms.

Three multivariate mixed effects logistic regression models were constructed to identify independent predictors for using government health care facility and for using private healthcare facility for illnesses in general, and for visiting any modern healthcare facility for TB-suspicious symptoms. In the first 2 models, worker occupation, duration of residence in Thailand and type of illness were retained within the model irrespective of statistical significance, and in the last model occupation, duration of residence and type of TB-suspicious symptom were similarly retained. Other variables were removed by backward elimination if not contributing significantly (i.e. if P > 0.05) to the fit of the model as evidenced using the likelihood ratio test. In each model the residence camp was considered as the random element and worker characteristics as fixed effects. This allowed adjustments to be made for the effects of intra-cluster correlation.

Estimates of the one-year period prevalence of having TB-suspicious symptoms in the period following the latest annual medical check-up among health-system-registered migrant workers were made using a mixed effects Poisson regression model. Variables independently associated with period prevalence were sought using this model.

### Ethical consideration

The research proposal was approved by Institute Ethics Research Committee of the Faculty of Medicine, Prince of Songkla University, Thailand. As the status of many respondents was insecure, full confidentiality of the records and anonymity of the workplaces included was strictly maintained. Prior to administration of the questionnaire, an introductory letter attached to the questionnaire was read to the participants in their own language, clarifying the purpose and voluntary nature of the study and summarizing issues of confidentiality, anonymity, informed consent and the importance of honesty in the response. The right to refuse participation was guaranteed. All participants received a small token gift following the interview. Respondents who were identified as having TB-suspicious symptoms were advised and encouraged to obtain access to appropriate care.

## Results

### Socio-demographic characteristics

A total of 614 migrant workers was enrolled in the study. The number of factory workers was slightly higher than planned due to some discrepancies in the reported numbers of workers in some factories prior to and during data collection. Only 10 persons refused to answer the questions. Table 1 presents the socio-demographic characteristics of respondents according to occupation. Of the workers surveyed, about 70% were males and 30% females among construction workers (CW), but with more males (77%) among rubber tappers (RT) and fewer males (63%) among factory workers (FW). Median age was 26 years in each occupation. Approximately 29% of FW and RT, but only 14% of CW could read and write only or were illiterate. Thirty-seven percent of FW and RT, but 43% of CW had primary school education, and 32% of FW, 26% of RT and 37% of CW had experienced middle school education. Only 1.4%, 6% and 8% of FW, RT and CW had high school education, and only one worker (FW) had graduated from a tertiary education institute.

**Table 1:** Socio-demographic variables of migrant workers by occupation

**Variable**	**FW**	**RT**	**CW**
	**Number (%)**	**Number (%)**	**Number (%)**
	**N = 214**	**n = 200**	**n = 200**
Age			
Mean (SD)	25.6 (7.1)	25.6 (7.11)	25.6 (7.1)
Sex			
Male	134 (62.6)	154 (77.0)	138 (69.0)
Female	80 (37.4)	46 (23.0)	62 (31.0)
Marital status			
Married	57 (26.6)	91 (45.5)	98 (49.0)
Single	134 (62.6)	80 (40.0)	87 (43.5)
Separated	14 (6.5)	17 (8.5)	12 (6.0)
Divorced	5 (2.3)	12 (6.0)	3 (1.5)
Widowed	4 (1.9)	0 (0.0)	0 (0.0)
Education status			
Illiterate	2 (0.9)	2 (1.0)	1 (0.5)
Read & write	60 (28.0)	57 (28.5)	27 (13.5)
Primary school	79 (36.9)	73 (36.5)	86 (43.0)
Middle school	69 (32.2)	52 (26.0)	74 (37.0)
High school	3 (1.4)	16 (8.0)	12 (6.0)
Graduate	1 (0.5)	0 (0.0)	0 (0.0)
Type of documents			
Border pass	0 (0.0)	0 (0.0)	0 (0.0)
Passport	39 (18.2)	0 (0.0)	1 (0.5)
Labour card	188 (87.9)	135 (67.5)	163 (81.5)
Health card	177 (82.7)	129 (64.5)	130 (65.0)
Social security card	0 (0.0)	0 (0.0)	0 (0.0)
Other documents	0 (0.0)	0 (0.0)	0 (0.0)

### Legal status

Regarding legal status, 18% of FW but only one CW and no RT had a passport. Among FW, RT and CW, respectively 88%, 68% and 82% held a labour card and 83%, 65% and 65% also held a foreign worker health card. None of the workers had a social security card. The foreign worker health card scheme allows migrants to access a government health care facility with a charge of 30 baht for each visit or admission. Only migrant workers who had a labour card were eligible to enroll in this healthcare scheme.

Among workers not having a labour card, approximately 17% of FW, 5% of RT and 49% of CW gave reasons related to financial problems. They needed to pay 1900 baht and had to check their health status to obtain a labour card. However, almost all of the workers without a labour card (100% of FW, 98% of RT and 97% of CW) also reported other reasons, including “waiting for the time that the government would call for labour cards”, “waiting for the time that the employers would arrange for them” and “having just arrived Thailand and not knowing how to go about obtaining a labour card”. No workers thought that the labour card was not useful. They all wanted to work in Thailand fearlessly.

On the other hand, among workers not having a foreign worker health card, 37% and 66% of FW and CW, but only 4% of RT reported the reason to be related to financial problems. For the health card, an extra 1900 baht was required for one year, whether or not they became ill during that year; thus 43%, 40% and 51% of FW, RT and CW, respectively, thought that the health card was not useful for them; 31%, 4% and 10% did not want to go to a government hospital, and 26%, 1% and 11% respectively preferred to use private clinics. Most of the workers (72% of FW and 94% of RT & CW) gave other reasons for not having health card, including “being busy”, “being rarely sick” and “the government hospital being too far away”. For factory workers, they could go to factory clinics for minor illnesses, and their employers already had arranged links with private hospitals for their major illnesses.

### General illnesses experienced while staying in Thailand

All workers had gone through at least one episode of illness during their stay in Thailand, especially common cold and aches and pains. Experience of GI problems was reported by 56%, 60% and 42% of FW, RT and CW, and respiratory problems by 86%, 81% and 52%, respectively. Among female FW, RT and CW, 40%, 61% and 57% had experienced obstetric problems (Table 2). Other health problems were usually minor injuries among FW and CW, but traffic accidents were reported by RT.

**Table 2:** Illnesses experienced by migrant workers while staying in Thailand by occupation

**Type of illness**	**FW**	**RT**	**CW**
	**Number (%)**	**Number (%)**	**Number (%)**
	**N = 214**	**n = 200**	**n = 200**
Common cold	214 (100.0)	197 (98.5)	179 (89.5)
Aches & Pains	212 (99.1)	197 (98.5)	200 (100.0)
GI problems	119 (55.6)	119 (59.5)	83 (41.5)
Respiratory tract problems	183 (85.5)	162 (81.0)	103 (51.5)
Urinary tract problems	49 (22.9)	76 (38.0)	68 (34.0)
Obstetric problems *	32 (40.0)	28 (60.9)	35 (56.5)
Gynaecologic problems *	58 (72.5)	21 (45.7)	21 (33.9)
Other health problems	214 (100.0)	197 (98.5)	197 (98.5)

* The totals of female FW, RT and CW were 80, 46 and 62, respectively.

### Healthcare-seeking behaviour for illnesses in general

Almost all workers (over 97%) employed self-medication for all types of illness except gastrointestinal and obstetric problems (Table 3). Care for GI problems was sought at a government health service facility, either hospital or health centre, by 8%, 35% and 66% of FW, RT and CW, and at private clinics by 45%, 81% and 28% respectively. FW made greater use of factory clinic (77%). However, for respiratory problems, all FW, 98% of RT and 97% of CW used self medication. None of the FW sought governmental healthcare for respiratory problems, only 2/162 RT and 12/103 CW (12%) with such symptoms went to government health services for treatment. On the other hand, of the female FW, RT and CW having obstetric problems, 16%, 89% and 94% had sought government healthcare service but very few had visited a private clinic. Of the FW who needed obstetric care, about half returned to their home country. For gynecologic problems, very few FW and RT but 8/21 CW (38%) had visited a government health facility, whereas 27/58 FW (47%) and 13/21 RT (62%) had visited a private clinic compared with only 2/21 CW (9%). Irrespective of occupation, between 50 and 60% of female workers with gynecological problems applied self medication using traditional medicine packets.

**Table 3:** Healthcare-seeking preferences among migrant workers for GI, obstetric and respiratory problems by occupation

**Health-seeking Preferences**	GI problems*			Obstetric problems*			Respiratory problems*		
	**FW**	**RT**	**CW**	**FW**	**RT**	**CW**	**FW**	**RT**	**CW**
	**No. (%) n = 119**	**No. (%) n = 119**	**No. (%) n = 83**	**No. (%) n = 32**	**No. (%) n = 28**	**No. (%) n = 35**	**No. (%) n = 183**	**No. (%) n = 162**	**No. (%) n = 103**
Government hospital	9 (7.6)	13 (10.9)	29 (34.9)	5 (15.7)	22 (78.6)	27 (77.1)	0 (0.0)	1 (0.6)	9 (8.7)
Government health centre	0 (0.0)	29 (24.4)	26 (31.3)	0 (0.0)	3 (10.7)	6 (17.1)	0 (0.0)	1 (0.6)	3 (2.9)
Factory clinic	92 (77.3)	0 (0.0)	0 (0.0)	0 (0.0)	0 (0.0)	0 (0.0)	9 (4.9)	0 (0.0)	0 (0.0)
NGO clinic	0 (0.0)	0 (0.0)	0 (0.0)	1 (3.1)	0 (0.0)	0 (0.0)	0 (0.0)	0 (0.0)	0 (0.0)
Private clinic	54 (45.4)	96 (80.7)	23 (27.7)	0 (0.0)	3 (10.7)	1 (2.9)	2 (1.1)	31 (19.1)	8 (7.8)
Self medication	23 (19.3)	45 (37.8)	37 (44.6)	1 (3.1)	1 (3.6)	1 (2.9)	183 (100.0)	158 (97.5)	100 (97.1)
Traditional healer	0 (0.0)	0 (0.0)	0 (0.0)	7 (21.9)	0 (0.0)	0 (0.0)	0 (0.0)	0 (0.0)	0 (0.0)
Others	0 (0.0)	0 (0.0)	0 (0.0)	16 (50.0)	4 (11.4)	0 (0.0)	0 (0.0)	0 (0.0)	0 (0.0)

The values of n in the column headings are the numbers of workers in each occupation who reported to have experienced the given health problem during their stay in Thailand.

### Factors related to seeking healthcare at government and private health service facilities

Factors independently associated with seeking healthcare at a government facility, identified by mixed effects logistic regression are shown in Table 4. Overall, FW were the least likely to use the government health service and CW the most likely. On the other hand CW were less likely than the other groups to use a private clinic. Having a foreign worker health card and, to a lesser extent having only a labour card, greatly increased the probability of using a government facility. Other factors increasing the odds of visiting a healthcare facility were being female (for government facility), and being married rather than single (for both government and private facilities).

**Table 4:** Mixed effects logistic regression model for seeking healthcare at a government and private healthcare facilities

**Variable**	**Category**	**Government healthcare facility**			**Private healthcare facility**		
		OR*	**95% CI**	**p-value#**	OR*	**95% CI**	**p-value#**
Current work	Rubber tapper	1^b^	0.01 – 0.16	<.001	1^b^	0.52 - 2.32	<.001
	Factory worker	0.04^a^	3.57 – 41.20		1.09^b^		
	Construction worker	12.14^c^			0.06^a^	0.24 - 0.16	
Legal status	No documentation	1^a^	1.26 – 66.38	<.001			
	Labour card	9.13^b^	11.52 – 325.5				
	Labour card + health card	61.22^c^					
Sex	Male	1	1.39 – 9.79	.006			
	Female	3.69					
Age group	0-20 yr				1^a^	1.20 - 3.99	.035
	21–30 yr				2.19^b^	0.81 - 5.79	
	31–100 yr				2.16^ab^		
Marital status	Single	1^a^	0.86 – 6.95	.009	1^a^	0.96 - 3.26	.047
	Separated/divorced/widowed	2.45^ab^	1.59 – 9.08		2.78^ab^	1.14 - 6.80	
	Married	3.78^b^			1.77^b^		
Duration of stay	Per year	1.17	1.05 – 1.31	.003	1.22	1.11 - 1.35	<.001
Illness	Common cold	2.1	0.34 – 13.1	.416	0.36	0.09 - 1.46	.170
	Aches and pains	0.48	0.03 – 9.01	.627	4.32	0.24 - 76.5	.319
	GI system	3.79	1.71 – 8.41	<.001	1.78	1.06 - 2.98	.028
	Respiratory system	4.06	1.60 – 10.33	.002	1.47	0.81 - 2.69	.207
	Urinary system	1.94	0.93 – 4.05	.069	1.05	0.62 - 1.77	.869
	Obstetric problems	20.58	5.51 – 76.9	<.001	0.97	0.48 - 1.94	.923
	Gynaecologic problems	0.52	0.15 – 1.76	.286	0.61	0.29 - 1.29	.192
	Other health problems				2.20	0.16 - 30.0	.536

# Likelihood ratio test.

Odds ratios within the same variable within each model not having a superscript in common differ significantly (P < 0.05, Wald test).

Compared with other illnesses, obstetric, gastrointestinal and respiratory problems were more likely to result in the workers seeking help at a government hospital, but only GI problems were more likely to bring workers to a private clinic. It was also found that the longer the workers stayed in Thailand, the more likely they were to have used one or other of the two types of health services facility.

### Reasons for choosing and not choosing government health services and private clinics

Among the workers who had sought care at government health services or private clinics, there were different kinds of reason for their choices. CW chose government services most, whereas RT chose private clinics most. The reasons of CW for preferring government health services were that government services were easily accessible (45%) and available at low cost (85%). On the other hand, RT also said that private clinics were easily accessible for them (95%) and had a short waiting time (45%). Of the 214 FWs, only 9% had used government health facilities, but 52% had gone to private clinics, because of the availability of factory clinics and the linkage between their factories and some private hospitals.

The major reason for not choosing either government or private health service was the perception that symptoms were not serious. Following that, government facilities were not utilized because of the perception that the service was “not available” (55% of RT and 44% of CW), “fear of police” (44% of RT), the “long distance to travel” (40% of FW) or “being too busy” (28% of FW and 21% of RT). Non-availability of service was not included among the reasons for not using private clinics because migrants could access private clinics as long as they had money. Private clinics were not utilized because of “fear of police” (60% of RT), “economic constraints” (23% of FW and 22% of CW), and “being too busy” (22% of FW).

Reasons for not utilizing the healthcare service that differed markedly between government and private facilities were “economic constraints” for private clinics and “long waiting time” for government facilities.

### TB-suspicious symptoms

Among symptoms that could be suggestive of tuberculosis during their stay in Thailand, cough for more than two weeks was reported by 16%, expectoration by 11%, low grade fever by 7%, haemoptysis by 3% and gradual loss of weight by less than 1% (Table 5). Altogether 183/614 workers (29.8%) had experienced at least one TB-suspicious symptom comprising 17%, 34% and 39% of FW, RT and CW, respectively.

**Table 5:** TB-suspicious symptoms of migrant workers by occupation

**TB-suspicious symptom**	**FW**	**RT**	**CW**
	**Number (%)**	**Number (%)**	**Number (%)**
	**N = 214**	**N = 200**	**n = 200**
Cough for >2 weeks	17 (44.7)	40 (58.8)	44 (57.1)
Expectoration	14 (36.8)	19 (27.9)	35 (45.5)
Haemoptysis	0 (0.0)	5 (7.4)	12 (15.6)
Gradual loss of weight	0 (0.0)	4 (5.9)	1 (1.3)
Low grade fever	7 (18.4)	17 (25.0)	17 (22.1)
Any TB-suspicious symptom	36 (17.8)	66 (34.0)	77 (38.5)

Among 495 registered migrants who had undergone an annual health check-up, 105 reported having experienced at least one TB-suspicious symptom in the period following the last check-up. Adjusting for the variable duration of the post-check-up period, the estimated one-year period prevalence of TB-suspicious symptoms following the annual check-up was 22.9% (95%CI 17.4% – 30.2%). However, the estimate was 4 to 5 times lower among FW than among RT and CW (Table 6). No other variable, apart from occupational group, was found to be associated with the period prevalence.

**Table 6:** Estimated one-year period prevalence of TB-suspicious symptoms following last health check-up by occupation

**Occupational group**	**One-year period prevalence (%)**	**95% CI**
FW	6.4^a^	3.5 – 11.8
RT	27.0^b^	19.3 – 37.8
CW	30.5^b^	21.8 – 42.5

Period prevalences not having a superscript in common differ significantly (P < 0.05, Wald Test).

### Healthcare-seeking behaviours for TB-suspicious symptoms

Around 90% of each occupational group used self-medication when developing TB-suspicious symptoms. No more than 5% of any group visited a government hospital despite 77% having a foreign worker health card (in addition to a labour card) and a further 8% having a labour card alone. Private clinics were visited by 43% of RT and by 13% each of FW and CW, and factory clinic was attended by 26% of FW when they developed TB-suspicious symptoms.

Among workers who used self-medication, traditional medicine alone was used by 12% and western medicine in prepackaged multidrug packets, with or without traditional medicine, by 88%. These multidrug packages were purchased at stalls run by vendors of same nationality. Of the workers having had low grade fever, 43% had taken prepackaged western medicine packets containing sulphadoxine-pyrimethamine to relieve their fever, and among those having had cough for more than 2 weeks, 26% had taken multidrug packets that included steroids, mostly dexamethazone.

### Factors associated with using any modern healthcare facility when experiencing TB-suspicious symptoms

Factors associated with seeking healthcare for TB-suspicious symptoms at any modern healthcare facility identified by mixed effects logistic model are shown in Table 7. People who did not use modern services used self and traditional medication. Construction workers were the least likely to seek health services to relieve TB-suspicious symptoms. Possessing both labour card and foreign worker health card increased the odds of using health facilities 6-fold. Education status was found to be associated with the outcome, in which workers with middle school education were more likely to access modern healthcare facility than those with higher educational attainment. Among TB-suspicious symptoms, low grade fever was more likely to result in the workers going to a modern healthcare facility for treatment.

**Table 7:** Mixed effects logistic regression model of healthcare-seeking at any modern healthcare facility for TB-suspicious symptoms

**Variable**	**Category**	**OR**	**95% CI**	**p-value#**
Current work	Rubber tapper	1^b^		.012
	Factory worker	0.86^b^	0.32 - 2.31	
	Construction worker	0.19^a^	0.07 - 0.52	
Legal status				
	No doc. + Labour card	1	1.57 - 20.27	.004
	Labour card + health card	5.63		
Education status	Illiterate/Read & write	1^ab^		.049
	Primary school	0.68^ab^	0.26 - 1.80	
	Middle school	1.84^b^	0.59 - 5.75	
	High school/Graduate	0.14^a^	0.02 - 1.02	
Duration of stay	Per year	1.13	1.01 - 1.26	.032
TB-suspicious symptoms	Low grade fever	9.58	2.97 - 30.86	<.001
	Gradual loss of weight	2.14	0.17 - 27.62	.569
	Haemoptysis	2.92	0.68 - 12.49	.152
	Expectoration	1.31	0.48 - 3.56	.591
	Cough for >2 weeks	2.62	0.90 - 7.62	.070

# Likelihood ratio test.

Odds ratios within the same variable not having a superscript in common differ significantly (P < 0.05, Wald test).

### Reasons for not choosing modern healthcare facilities or private clinics for TB-suspicious symptoms

**Figure 1:**
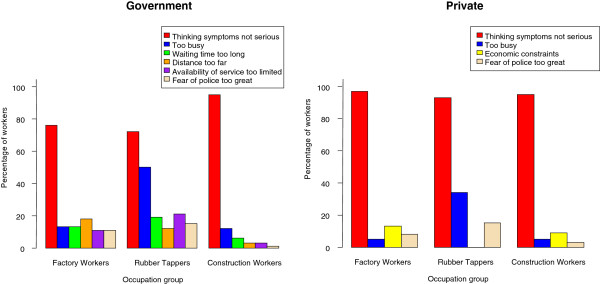
Reasons for not using government or private healthcare facilities for TB-suspicious symptoms.

As with other illnesses, the major reason for not visiting a modern healthcare facility for TB-suspicious symptoms was the perception that the symptoms were not serious (Figure 1).

Other reasons for not visiting a government facility, reported by between 13% and 18% of FW, were “being too busy”, “having to wait too long”, “the distance being too far”, “the services too limited” and “fear of police”. These reasons were reported by similar proportions of RT except that they more frequently reported “being too busy” (50%). Almost 100% of CW gave the reason of “symptoms being not serious” and only a small number mentioned the other reasons, among them “being too busy” was the most prominent (12%).

For not visiting a private clinic, “being too busy” was most commonly reported by RT (34%) but by less than 5% of both FW and CW. “Economic constraints” was the reason given by 13% of FW and 9% of CW but not by any RT. “Fear of police” was reported most commonly by RT (15%) but also by small numbers of FW (8%) and CW (3%).

## Discussion

The hypothesis that healthcare-seeking behaviours of foreign migrant workers in Thailand differ among different occupational groups was supported. Nevertheless, in all three occupations, self-medication was the most common way of dealing with most illnesses, including the development of TB-suspicious symptoms. Only for GI symptoms and obstetric problems did migrant workers commonly seek healthcare at modern healthcare facilities. The predominance of self medication has been similarly noted in a review of health service utilization among ethnic minorities [9] as well as in many other studies of international migrants in developed countries [10,11] and of internal migrants in China [12]. In UK, self-medication was described as one of the main barriers hindering migrants from accessing primary healthcare services, even though the respondents there were well educated [10], in contrast to those in our study, who mostly had no more than middle-school education, being low-skilled workers.

For GI problems, workers of all three occupations made greater use of modern healthcare facilities, although not to the exclusion of self-medication. Private facilities were greatly preferred over government health services by FW and RT but government facilities were preferred by CW. Easy access and short waiting time were reasons for using, and economic constraints reasons for not using, private clinics. An earlier study in Thailand reported similar reasons for the preference of migrants for private clinics, namely convenience in terms of time, legal status and transportation [13]. Among the information provided by our respondents was the observation that private service providers never asked them for their identification card or about their background so that they did not need to worry about their legal status.

By contrast, for obstetric problems, especially delivery, government health facilities were overwhelmingly preferred over private facilities by female migrants in all three occupations, although half of the female FWs returned to their home country when they were about to give birth. Qualitative information obtained in our study suggests that the FW commonly returned to their home country for delivery because they were forced to quit their job when they became pregnant, but could get their job back after their delivery.

Unlike RT and CW, FW employed in large factories were able to attend in-factory clinics to obtain treatment for minor ailments, while for more severe illnesses the factory would arrange for them to attend at large private clinics with which they had pre-existing arrangements. Thus, there was little need for FW to attend government hospitals or clinics. RT also made little use of government healthcare services, as their generally wealthier status meant that the higher cost at private clinics did not outweigh the added convenience of rapid service and freedom from worry over their often precarious legal status. CW shared neither the higher economic status of the RT nor the accessibility of a work-place clinic or factory-arranged referral to a private clinic, and this probably explains their relatively greater utilization of government healthcare providers for GI problems. The differential was shown clearly after adjustment for confounding variables in the regression models, in which CW were much more likely than workers in the other occupations to avail themselves of government services and far less likely than RT to utilize private hospitals or clinics. Economic constraints have similarly been reported in other migrant populations to be determinants of healthcare behaviour [9,12,14]. Affordability may also explain the greater preference of female than of male and of married than of single migrant workers to utilize government healthcare services. Our qualitative information indicated that female workers were more concerned about saving money, while married workers may have higher expenses for daily living.

The proportion of workers reporting having experienced TB-suspicious symptoms during their stay in Thailand, around 30%, is likely to be an underestimate of the true proportion. Most of the migrant workers were seemingly reluctant to answer admit to having had such symptoms. A review of the TB situation compiled by the Government of Thailand and WHO mentions that less than 50% of the estimated number of TB cases were notified even among the indigenous population [15]. In fact, the migrant population is likely to be at greater risk of tuberculosis because most of the workers were young (median age of 25.6) and male (69%), and tuberculosis has been reported to be more likely to occur among males of 15–54 yrs of age [16]. Furthermore, tuberculosis is deeply rooted in populations where human rights and dignity are limited [6].

Our estimates of the one-year period prevalence of TB-suspicious symptoms following the last annual health checkup indicate a considerably higher prevalence among CW and RT than among FW. The difference might be explained by the generally poorer conditions of the accommodation of RT and CW, a finding that has also been reported among both migrants and non-migrants in other settings [9,17]. However, the high prevalence of TB-suspicious symptoms among CW might also be attributed partly to the unfavourable dusty environment of construction sites, which may render CW vulnerable to cough or expectoration.

As with most other health problems, self medication of TB-suspicious symptoms was extremely common, and the drugs used were inappropriate. Such workers preferred to use prepackaged western medicines over traditional medicines. This practice mirrors that reported among TB suspects in other studies [18,19], including a survey among Myanmar migrant workers in different region of Thailand, in which around half of the workers failed to get any treatment until their health deteriorated considerably [13].

Overall, only about 1 in 4 workers with these symptoms visited a modern health facility, usually a private or factory clinic. CW were the least likely to visit a modern healthcare facility for these symptoms, even after adjusting for the possession of a health card and/or labour card. It was surprising that having an education above middle school was associated with a lower probability of attending a modern healthcare facility when TB-suspicious symptoms developed. No satisfactory explanation has been found.

Undetected TB cases could be present among these TB suspects. The common practice of self-medication for symptoms suggestive of TB without seeking any treatment at an appropriate healthcare service is likely to prevent cases being diagnosed correctly as pulmonary TB. This could affect the case finding of the National TB Programme. Indeed, high prevalences of pulmonary TB have been reported among TB suspects who had suspicious symptoms in other settings [17,20].

Workers in our study frequently bought prepackaged western medicine from vendors of the same nationality. Drug stores are often the first and only, or preferred, source of healthcare outside home for a majority of patients in developing countries [21,22]. It was striking in our survey that 41% of the workers having low grade fever took prepackaged packets containing sulphadoxine-pyrimethamine to relieve their fever. Sulphadoxine-pyrimethamine is in fact an antimalarial drug. Misuse of antimalarial drug can result in drug resistance to malaria and frustrate measures to effectively manage the disease as drug resistance will lead to treatment failure [23,24]. The problem may be greatest among CW, who were found to have a high prevalence of TB-suspicious symptoms and yet utilized modern health care facilities the least.

Inappropriate use of glucocorticosteroids in multidrug packets was also investigated among the workers having cough for more than two weeks. When they went to the vendors for cough, they were given the specific combination containing dexamethazone, which is a kind of glucocorticosteroid. Because glucocorticoids are commonly used immunosuppressive agents, their impact on the risk of tuberculosis is important. In one study in UK, patients who were exposed to a glucocorticoid had an approximately 5-fold increased risk for developing new tuberculosis, independent of other risk factors [25]. Amongst patients with systemic sclerosis on dexamethazone-pulse therapy, it was concluded that there was an increased risk of tuberculosis [26]. Therefore, the respondents of our survey with cough for more than 2 weeks, a well-known TB-suspicious symptom, could progress from subclinical tuberculosis to active disease because of using dexamethazone inappropriately. Workers usually took these dexamethazone-containing packages two to three times a day for at least five days continuously to relieve their prolonged cough.

The reasons given by our respondents for using self medication and not trying to seek professional treatment for their TB-suspicious symptoms were variable. All three types of worker in our survey similarly thought that these symptoms were not serious enough to seek professional treatment. They were waiting to see whether or not their symptoms would become severe. Other studies also found that TB suspects preferred to wait and see whether their symptoms would become serious as they did not want to pay high healthcare costs out of pocket unnecessarily [18,19]. RT in our study did not worry about spending money as they earned more than FW and CW, but they were also the busiest workers and could less readily afford the time to attend at a modern healthcare facility. They worked at night and had to sleep in the daytime. A WHO report similarly notes that people with a full time job are usually too busy to seek professional healthcare, especially when they think their symptoms are not severe enough to justify stopping their work and seeking treatment [19].

Our study has a number of strengths. The respondents were interviewed in their own language by interviewers of the same nationality, thereby facilitating rapport. Within each camp, the subjects were selected randomly and with a constant sampling fraction within each camp so that, despite the snowball sampling technique to choose the camps, the study sample should have been well representative of the chosen camps within each occupational group. The migrant workers were interviewed at their own residence so that they felt safe and could respond well to the questions.

Nevertheless, the study also has some limitations. Firstly, snowball sampling to locate and select workers’ camps for inclusion could potentially lead to a sample that is not fully representative of the population in general. Unfortunately, in the absence of complete enumeration lists of migrant workers, particularly those who have questionable legal status, we could not avoid using this non-random method as the first stage of sampling. Snowball technique is the simplest approach to reach hidden populations. Secondly, the healthcare-seeking behaviours of the workers in the same camp were likely to be similar as they lived very close to one another. This problem was minimized by using mixed effects regression modeling in the analysis, which adjusted the estimates of coefficients for clustering on camp. Thirdly, the information on history of illness and healthcare-seeking behaviour was based on recall and perception of the interviewees and not independently verified with data from the formal healthcare facilities. Thus, the data obtained may not have been a totally valid representation of actual illness and healthcare-seeking behaviour. Nevertheless, the aim was to identify healthcare-seeking behaviours of the migrants when they perceived themselves to be ill so, in this context, their actual health status was not the prime concern. Lastly, it is not certain to what extent the healthcare-seeking behaviours described in the study are attributable to migrant status *per se*, as a comparison group of similar host country workers or of workers in the country of origin was not included. As migrant workers in the three occupations included have almost replaced host country workers and access to study workers in the migrants’ home country was not permitted, no comparable group was available.

## Conclusions and recommendations

In conclusion, the study contributes to our understanding of the different patterns of healthcare-seeking behaviour among migrant workers in the three different occupations in Thailand. Despite their not being independently verified against actual health status, most perceived illnesses were treated by self medication alone, apart from GI and obstetric problems. Health is a priority only when the migrant workers feel sick; otherwise issues around immigration and their employment take precedence. There appears to be a need to improve the health situation, risk perception and healthcare-seeking behaviour of the migrants. This might be facilitated by solving the current problems and removing the perceived barriers to health service access. Overall, CW appear to be most in need of improved access to healthcare.

Several recommendations can be made from the findings of this study. Self medication is the most widely adopted healthcare-seeking behaviour for most illnesses, including the occurrence of TB-suspicious symptoms. The attendant problems, such as delay in seeking professional care and possible adverse health effects of the self-administered medications themselves, need to be addressed.

In the setting of the current study, local stalls run by vendors of the same nationality as the migrants were major sources in the inappropriate self medication by workers with TB-suspicious symptoms. These vendors have the potential for playing a role in facilitating the detection of TB cases. In our study, these vendors were found to be easily trainable and to be very familiar with the workers in their catchment communities. Leaflets in their mother tongue regarding health education about TB could be distributed to foreign migrant populations via these vendors in order to provide TB information. Some studies have shown that information leaflets can be used to improve knowledge [27]. This improvement could contribute to early diagnosis and prompt treatment and thereby limit further spread.

Active case finding among the migrant workers with TB-suspicious symptoms should also be intensified by the National TB Programme. Worldwide, NTPs practise passive case finding as the standard strategy for case detection [6]. Active case finding could find hidden smear-positive TB cases [28]. Detection of TB cases among the migrant TB suspects would lead to early diagnosis and thereby to a shortening of the duration of infectiousness before DOTS is initiated. This may be particularly important among CW, among whom the period prevalence of TB-suspicious symptoms is high but attendance at modern healthcare facilities particularly low.

## Abbreviations

FW: Factory workers; CW: Construction workers; GI: Gastrointestinal; NGO: Non-governmental organization; RT: Rubber tappers; TB: Tuberculosis; WHO: World Health Organization.

## Competing interests

The authors declare that they have no competing interests.

## Authors’ contributions

All authors participated in the conception and design of the study. TN undertook data collection. TN and AG undertook statistical analysis of the data and together with PP interpreted the findings. All authors contributed to the preparation of the manuscript and have read and approved the final version.

## Pre-publication history

The pre-publication history for this paper can be accessed here:

http://www.biomedcentral.com/1472-698X/12/22/prepub
